# Occupational Therapy Interventions in Adults with Multiple Sclerosis or Amyotrophic Lateral Sclerosis: A Scoping Review

**DOI:** 10.3390/ijerph18041432

**Published:** 2021-02-03

**Authors:** Luis De-Bernardi-Ojuel, Laura Torres-Collado, Manuela García-de-la-Hera

**Affiliations:** 1 Department of Public Health, History of Medicine and Gynecology, University Miguel Hernández, 03550 Alicante, Spain; luisdebernardiojuel@gmail.com (L.D.-B.-O.); manoli@umh.es (M.G.-d.-l.-H.); 2 Spanish Consortium for Research on Epidemiology and Public Health (CIBERESP), 28029 Madrid, Spain; 3 Alicante Institute for Health and Biomedical Research, ISABIAL-UMH, 03010 Alicante, Spain

**Keywords:** occupational therapy, multiple sclerosis, amyotrophic lateral sclerosis, intervention

## Abstract

This scoping review aims to describe occupational therapy interventions carried out with multiple sclerosis (MS) and amyotrophic lateral sclerosis (ALS) patients in occupational therapy. A peer review of the literature was conducted in different databases: Pubmed, Scopus, Web of Science and Embase, and in some occupational therapy journals. A search of the literature published was carried out before December 2019. The inclusion criteria were as follows: (1) articles evaluating the intervention of occupational therapy in MS or ALS including experimental, randomized, nonrandomized and exploratory studies; (2) written in English or Spanish; (3) adult population (over 18 years old). The initial search identified 836 articles of which we included 32 divided into four areas of intervention: fatigue-targeted interventions, cognitive interventions, physical interventions and others. Only 16 studies were carried out exclusively by occupational therapists. Most occupational therapy interventions are aimed at fatigue and physical rehabilitation. The majority of the studies in our review included MS patients, with little representation from the ALS population. These interventions have shown an improvement in perceived fatigue, manual dexterity, falls prevention and improvement in cognitive aspects such as memory, communication, depression and quality of life in the MS and ALS populations.

## 1. Introduction

Multiple sclerosis (MS) and amyotrophic lateral sclerosis (ALS) are neurodegenerative diseases of the nervous system [1,2]. These diseases have a medium to high prevalence, but recently published epidemiological studies have shown an increasing incidence and prevalence of MS and ALS in different populations worldwide [3,4,5]. Globally, in the case of MS, the incidence is 2.5 per 100,000 inhabitants, while in ALS it is between 0.6 and 3.8 per 100,000 inhabitants, although the age of onset is later than in MS [3,6]. Though the origin of the diseases is unknown, previous studies have ruled out risk factors such as geographical latitude or ethnicity [7], while other studies suggest that having a family member with these diseases can increase the risk of developing them [8]. However, the mechanisms and causes for their development are not completely understood.

These diseases affect not only quality of life but also physical and cognitive aspects, increasing fatigue and the probability of suffering from depression [9]. They can include multiple symptoms such as muscle stiffness, paralysis of the lower and upper limbs, sensory dysfunction, visual problems, ataxia, dysarthria or dysphagia [10] as well as cognitive impairment and psychological problems in the affected persons [2,11,12]. These diseases have a multidimensional impact on a person´s life, and their symptoms imply a significant loss of autonomy which greatly affects their occupational performance [11].

The treatment of these diseases is carried out by multidisciplinary teams [11,13] and it can be pharmacological [14] and/or nonpharmacological. In nonpharmacological treatment, there are specific interventions aimed at physical rehabilitation such as electro-stimulation or Proprioceptive Neuromuscular Facilitation (PNF) [15]; there are also interventions to maintain daily life skills and others aimed at psychological or cognitive rehabilitation with the participation of occupational therapist [11,13]. Regarding cognitive intervention, Lincoln et al., carried out a comparative study based on attention and memory which resulted in an improvement in memory and lifestyle in the experimental group compared to the control group [16]. 

Some previous occupational therapy studies have proposed psychosocial promotion interventions [17,18] such as the Community Reintegration for Socially Isolated Patients (CRISP) occupation-based intervention, which used education and self-management strategies in MS patients, performing socializing and recreation activities to improve self-efficacy and to reduce perceptions of loneliness [18]. Other studies have proposed cognitive rehabilitation interventions to maintain everyday tasks [19], meal preparation and finance management [20]. Finally, it should be pointed out that the majority of the studies led by occupational therapist in the treatment of MS were based on Packer and colleagues’ fatigue management intervention. These studies were carried out for both inpatients [21] and outpatients [22] and they all sought to maintain or improve the patients´ occupational performance and quality of life, and to improve muscle strength, energy levels as well as other more cognitive aspects [11]. 

MS and ALS have a significant impact on people´s activity and participation [11,12]. Recent reviews showed that occupational therapy could improve occupational performance and other outcomes in MS and ALS populations [23,24]. Nevertheless, these reviews focused on the effectiveness of occupational therapy intervention and contained little information about the activities performed and the role of occupational therapists in the intervention. In this sense, several key gaps in the literature impair a complete understanding of how all previously published interventions in MS and ALS with the participation of occupational therapists, were carried out. We would like to underline that this scoping review provides occupational therapists with tools to perform evidence-based interventions, due to an updated summary of previous evidence that exists on MS and ALS interventions. Thus, we aim to describe those interventions carried out with MS and ALS patients in occupational therapy. In particular, we want to answer the following question: Which interventions are performed from occupational therapy in adult people with MS or ALS?

## 2. Materials and Methods 

A search of the literature published before December 2019 was undertaken by two independent reviewers following the recommendations of the Cochrane Manual [25], the Joanna Briggs Institute [26] and PRISMA Extension for Scoping Reviews (PRISMA-ScR) [27]. The same strategy and key words were used in the different bases: “occupational therapy” and “intervention” and “sclerosis” (Table 1).

We searched the literature in 4 databases: Pubmed, Scopus, Web of Science and Embase, and in first-quartile Occupational Therapy scientific journals according to Scimago journal rank in 2018 (American Journal of Occupational Therapy, Journal of Occupational Rehabilitation, Physical & Occupational Therapy in Pediatrics and OTJR: Occupation, Participation and Health). In addition, we performed a search in the grey literature in TESEO to identify possible unpublished studies.

The inclusion criteria were as follows: (1) articles evaluating the intervention of occupational therapy in MS or ALS including experimental, randomized, nonrandomized and exploratory studies; (2) written in English or Spanish; (3) adult population (over 18 years old). We excluded the following: (1) qualitative studies; (2) studies with no abstract, no full text or not available.

Study selection and data extraction of the information were carried out independently. We migrated the results from the databases to a Microsoft Excel spreadsheet where inclusion and exclusion decisions were recorded. The two reviewers (LB and LT) independently selected articles based on the selection criteria. Any disagreement between them regarding possible inclusion/exclusion criteria was resolved by a third reviewer (MG). LB and LT only had discrepancies regarding the inclusion of one article, and with the intervention of a third reviewer we decided to exclude it. 

## 3. Results

**Figure 1:**
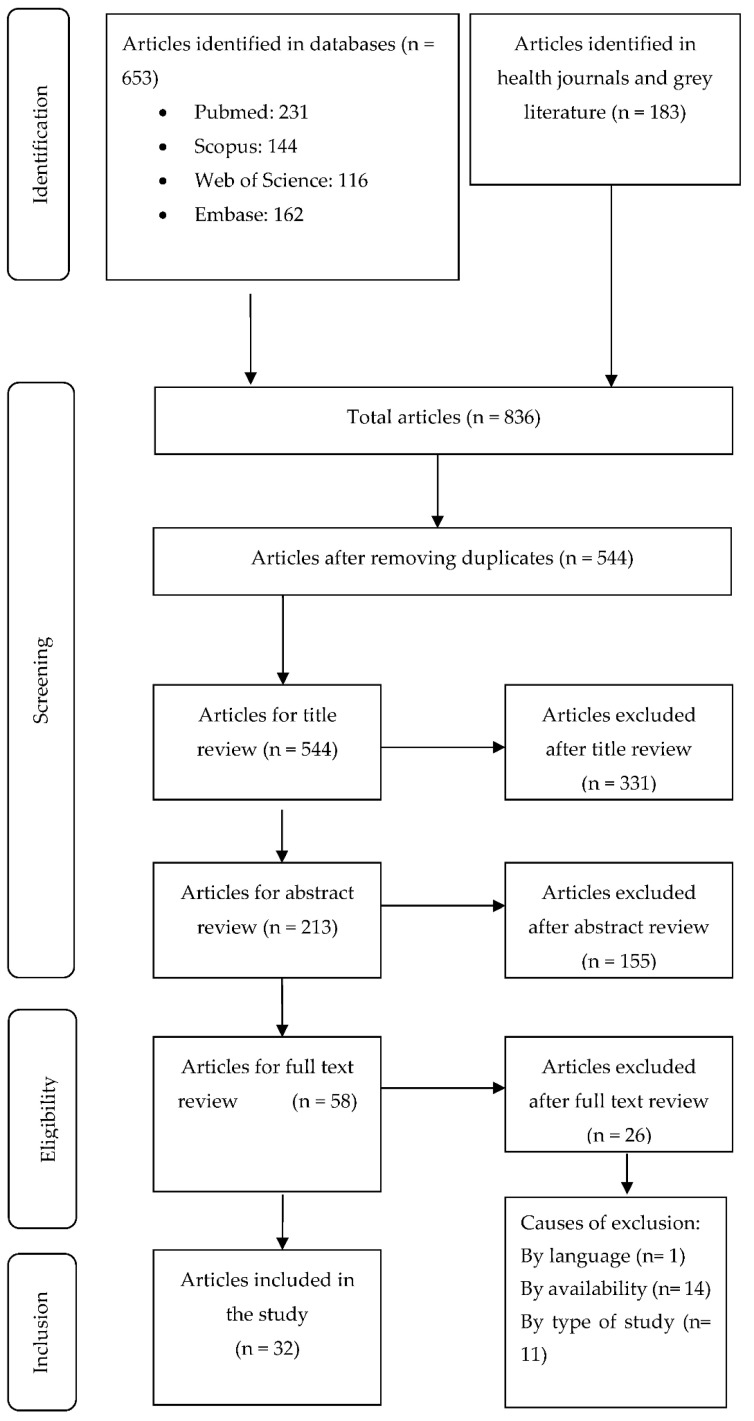
Flowchart of the study selection process.

The search strategies identified a total of 836 articles and, after conducting the peer review, 58 articles were selected for their retrieval and evaluation of the full text. We excluded 26 articles, as they did not fulfil the inclusion criteria, leaving 32 articles for data analysis and extraction. The flow chart is shown in Figure 1.

The 32 selected studies were conducted in different countries: in USA (*n* = 12), Belgium (*n* = 4), Netherlands (*n* = 3), Switzerland (*n* = 3), Spain (*n* = 2), Italy (*n* = 2), and the remaining 6 studies were conducted in Cuba, Ireland, United Kingdom, Israel, Iran and Austria. A total of 29 studies were carried out in MS patients and only three studies were carried out in ALS patients. A total of 16 studies were led exclusively by occupational therapists and the remaining studies were carried out by multidisciplinary teams, including neurologists, neuropsychologists, social workers or experts in certain fields such as assisted technology or mathematics. 

The main limitations reported by included studies were small sample size, lack of long-term evaluation of the intervention, lack of randomization and low generalizability of the results. Table 2 presents the characteristics and information of the included studies: author, year, country where study was conducted, objective of study, sample, intervention, standardized proof used, main results and conclusions of study following Cochrane Manual recommendations [25]. 

We observed that intervention studies conducted in MS and ALS patients could be classified into four clearly differentiated areas: fatigue, physical rehabilitation, cognitive interventions and others. The full description of these interventions are presented in Table 3. 

The articles analyzed were classified into four clearly differentiated areas: (1) interventions for fatigue and energy conservation; (2) cognitive interventions; (3) physical interventions; and (4) other interventions. The fully description of OT intervention carried out in MS and ALS is shown in Table 3.

### 3.1. Interventions in Fatigue and Energy Conservation

Twelve studies conducted interventions related to energy and impact on fatigue in people with MS. The results of these interventions are described in Table 2. Seven of these studies were based on the fatigue management program developed by Packer et al. [28], which consists of a 12 h intervention for people with MS and includes a balanced lifestyle, rest, posture and efficient communication, among other aspects [29,30,31,32,33,34,35]. Another study modified Packer´s program and evaluated these interventions nonpresentially by monitoring patients either through teleconferences, applications or on the internet [36].

In addition, several authors examined the effectiveness of physiotherapy and diet interventions aimed at fatigue carried out by a multidisciplinary team, including occupational therapists [37,38]. Other authors proposed different intervention programs [39,40] based on changes in daily occupational performance and proposed strategies related to occupational balance, activity, fatigue, energy account, goals or effective communication.

We did not identify studies carried out in fatigue and energy conservation in ALS patients.

### 3.2. Cognitive Interventions

Six studies carried out cognitive interventions. A full description intervention appear in Table 3.

Of these studies, two included the use of technology to facilitate communication and automated control at home [19,41] to evaluated the functional performance which increased significantly with PDA use [18]. The remaining studies evaluated interventions related to improvement of memory, attention, processing speed and strategies to compensate these cognitive strategies [20,42,43,44] with different results (Table 2)

Only one study was carried out in people with ALS [41]. This study evaluated the feasibility and usability of an assistive technology prototype in users who have different degrees of muscular impairment to improve interaction with environment.

### 3.3. Physical Interventions

Of a total of ten articles describing interventions in relation to physical condition, nine focused on MS patients and one on ALS patients, only 2 were led by occupational therapists. The results and the details of interventions are shown in Table 2 and Table 3.

In the MS focused studies, we identified three categories: upper limb recovery, physical rehabilitation and falls prevention.

In the first category, four of the studies focused on upper limb recovery, both at the level of sensory re-education and at that of improvement in manual dexterity in MS [45,46,47,48]. Another study assessed an intervention program to improve the physical resistance of MS patients [49].

In the second category, two other MS focused studies aimed at physical rehabilitation were carried out using new technologies such as virtual reality [50] or images and videos [51].

Finally, the third category included two intervention studies which evaluated programs to decrease falls risk in MS patients, by sending them information related to falls and how to avoid them [52] or by giving them tape training sessions in order to improve balance reactions [53].

In relation to the ALS focused study, Gómez-Fernández et al., assessed the effectiveness of a multifactorial program by working on different aspects such as breathing, posture control or transfers using a multidisciplinary approach [54] which showed that ALS patients improved significantly on forced vital capacity.

**Table 2:** Main characteristics of the studies included in the review.

Authors, Year, Country	Objective	Sample (*n*), Disease	Intervention	Results	Conclusions
Eyssen et al. [55], 2013, Netherlands	To evaluate the effectiveness of a client-centred occupational therapy.	269, MS	Client-centred occupational therapy	The IG results were not significant and in the second measuring results were negative.	There was no improvement in disability, participation and autonomy in IG.
Eyssen et al. [56], 2014, Netherlands	To check whether client-centred practice spends more time on assessment than on intervention.	269, MS	Client-centred occupational therapy	The results showed a significant increase in time dedication on the diagnostic process in the IG.	The client-centred practice devotes too much time to the evaluation process with no improvements.
Block et al. [57], 2009, United States	To evaluate the effectiveness of the development of capacities and the health promotion in self-efficacy and ability to achieve objectives.	35, MS	Health promotion in self-efficacy and empowerment	The results showed significant improvements in self-efficacy and ability to achieve objectives.	The program could took action in multiple areas of intrapersonal, interpersonal, and behavioral functioning.
Raglio et al. [58], 2016, Italy	To evaluate the effectiveness of a music therapy and its influence on anxiety, depression or QoL.	30, ALS	Music therapy	There were only improvements in Mc Gill Quality of life Questionnaire.	The music therapy program showed an improvement in the QoL.
Reilly y Hynes. [42], 2018, Ireland	To evaluate the efectiveness of an occupation-based cognitive program in improving daily life and cognitive decline.	12, MS	Cognitive intervention (CI) for managing employment and daily life.	There were significant improvements in all areas.	CI is considered the most appropriate intervention. It can be more effective in newly diagnosed people.
Chiaravalloti et al. [43], 2018, United States	To examine the efectiveness of a SPT.	21, MS	Cognitive intervention in SPT.	The group that received SPT obtained better results than the CG in processing speed, learning and memory, and performance.	Results provide support of SPT in treating processing speed deficits in persons with MS.
Goverover et al. [20], 2017, United States	To examine the effectiveness of a self-generated program of memory and learning strategies.	35, MS	Cognitive intervention to improve memory and learning	The IG improved learning, memory, self-regulation, metacognition, depression, functional status, and QoL.	Results provides evidence that the intervention improves memory and affective symptomatology.
Schettini et al. [41], 2015, Italy	To evaluate the feasibility and usability of an assistive technology prototype for communication.	8, ALS	Cognitive intervention in usability of an assistive technology prototype for communication and home control	There were no significant differences between the different measures.	The study shows the feasibility and usability of assistive technology prototype.
Gentry. [19], 2008, United States	To evaluate the effectiveness of a PDA training program, as the assistive technology.	21, MS	Cognitive intervention with de use of PDAs to improve occupational performance.	Functional performance increased significantly with PDA use.	PDA still work as a compensatory measure for their deficit in executive functions, but it does not improve memory.
Shevil et al. [44], 2009, Israel-United States	To increase knowledge of cognitive impairments, increase levels of self-efficacy and increase use of management strategies.	35, MS	Cognitive intervention with a program (Mind over Matter) for the knowledge and management of the cognitive deficits.	Participants significantly increased knowledge of cognitive impairments and levels of self-efficacy in their ability to manage cognitive difficulties.	The results support benefits of self-management cognitive perspective to improve cognitive symptoms.
Gómez-Fernández et al. [54], 2001, Cuba	To examine the effect of multifactorial treatment in health.	6, ALS	Multifactorial physical intervention with intensive rehabilitation programme.	People improved significantly on forced vital capacity and Functional Rating Scales.	Multifactorial rehabilitation works well for the health and survival.
Yang et al. [53], 2019, United States	To explore if patients can adapt to imbalances after a program of training in falls on a treadmill.	13, MS	Physical intervention to improve stability and falls prevention.	There was a significant reduction in falls and significant improvements in stability and position.	With this training, people with MS may be able to improve their postural adjustments to prevent falls.
Kamm et al. [46], 2014, Switzerland	To evaluate the effectiveness of home-based program to improve manual dextery.	39, MS	Physical intervention with manual dextery training.	People improved significantly manual dextery and no significant differences in strength straining	Home manual dextery training improved fine mobility in relation with activities of daily living.
Lamers et al. [45], 2019, Belgium	To evaluate the effectiveness of a task-oriented upper limb program.	20, MS	Physycal intervention with task-oriented upper limb training by individualizing the intensity of training.	There were significant improvements of Action research arm test, Manual Ability Measure-36.	All participants performed the task-oriented training at their individualized intensity without any adverse effects.
Finlayson et al. [52], 2009, United States	To evaluate “Safe at Home BAASE” program for the management of falls risk.	30, MS	Physical intervention with the “Safe at Home BAASE” program.	Significant improvements in knowledge, prevention and manage of falls risk with 5:6 sessions.	The program has potential to improve knowledge, skills and behavior associated with reduced personal fall risk.
Ortiz et al. [50], 2013, Spain	To examine postural control and balance with a virtual reality telerehabilitation program.	50, MS	Physical intervention with a telerrehabilitation program to improve balance and postural control.	Significant improvement in balance, visual preference, the contribution of vestibular information, mean response time and Tinetti test yielded.	The rehabilitation program with virtual reality could be an alternative to standard rehabilitation programmes.
Waliño-Paniagua et al. [47], 2019, Spain	To compare the conventional occupational therapy treatment by virtual reality in manual dexterity training.	16, MS	Physical intervention with virtual reality training in manual dexterity.	Program showed no significant differences in manual dexterity. Improvements were found in precision, execution times, and the efficiency of functional tasks.	This therapy with virtual reality can be complementary to conventional intervention.
Bovend´Eerdt et al. [51], 2010, United Kingdom	To evaluate the effectiveness of a motor imagery program compared with OT.	30, MS	Physical intervention with a motor imagery program.	Compliance with advised treatment was poor in 85% of the therapists and in 72% of the patients.	Therapist and patient compliance was low, restricting the conclusions of the effectiveness of the imagery program
Kalron et al. [48], 2013, Israel	To evaluate the effectiveness of a sensory home-based hand re-education and manual dextery program.	18, MS	Physical intervention with a sensory hand re-education and manual dextery program.	There were significant improvements in the IG on nine-hole peg test and functional dexterity test.	Sensory re-education training at an early stage of the disease can slow the progression of manual dexterity deterioration.
Bansi et al. [49], 2013, Switzerland	To investigate the impact of endurance training in health-related quality of life and fatigue	60, MS	Physical intervention with endurance training with cycle-ergometer/aquatic-bike on QoL and fatigue.	Endurance training affects QoL and fatigue. Cardiorespiratory fitness and short-term TH2 were associated with better QoL.	Endurance training impacts on QoL and fatigue independently of the type of training (cycle-ergometer or aquatic-bike).
Vanage et al. [29], 2003, United States	To evaluate the effectiveness of an energy conservation program on fatigue.	37, MS	Energy conservation program	Fatigue was and physical, cognitive, and psychosocial measures were improved.	The program was effective and reduced levels of fatigue in people with moderate-severe MS.
Rietberg et al. [36], 2014, Netherlands	To evaluate a multidisciplinary rehabilitation program for chronic fatigue compared to a nursing consultation program.	48, MS	Multidisciplinary fatigue intervention	There were no significant differences in most fatigue measures.	Multidisciplinary rehabilitation was not more effective in reducing self-reported fatigue compared to nurse consultation.
Mathiowetz et al. [30], 2001, United States	To evaluate the efectiveness of an energy conservation program for its impact on fatigue, self-efficacy and quality of life.	54, MS	Energy conservation program	Participants reported significantly less fatigue impact, increased self-efficacy, and improved quality of life.	The energy conservation program is effective in improving fatigue.
Ghahari et al. [34], 2009, Iran	To evaluate the effectivenes of a fatigue self-management program.	23, MS	Fatigue intervention with an online self-manegement program	Participants exposed to pilot 3 (forums, activities online and quiz) improved significantly on the fatigue impact scale.	The results show that the online fatigue self-management program is a viable complex intervention.
D´hooghe et al. [36], 2018, Belgium	To evaluate the feasibility of a TeleCoach progam for the improvement of physical activity and fatigue levels.	75, MS	Fatigue intervention with TeleCoach program through smartphone.	There were significant improvements in Fatigue Scale for Motor and Cognitive Functions.	The TeleCoach program is viable as complementary training to conventional treatment.
Kos et al. [38], 2007, Belgium	To evaluate the effectiveness of a fatigue management program.	51, MS	Multidisciplinary fatigue intervention.	A reduction of Modified Fatigue Impact Scale was found in 17% of IG compared to 44% after the placebo intervention programme	The multidisciplinary fatigue management programme showed no efficacy in reducing the impact of fatigue compared to a placebo intervention programme
Sauter et al. [35], 2008, Austria	To examine the effectiveness of fatigue management and energy conservation strategies.	32, MS	Fatigue intervention	Significant improvements were found in people’s physical and cognitive fatigue. There were less fewer signs of depression and the quality of sleep improved	Fatigue cannot be completely eliminated, but there were improvements in fatigue management and energy conservation
Mathiowetz et al. [32], 2005, United States	To evaluate the effectiveness of an energy conservation course.	169, MS	Energy conservation program	There were significants effects on reducing the physical and social subscales of fatigue and on increasing QoL.	The energy conservation program is effective in improving fatigue, self-efficacy and quality of life in people with moderate-severe MS
Finlayson et al. [33], 2011, United States	To evaluate the effectiveness of a teleconference-delivered program on fatigue management	181, MS	Fatigue intervention through teleconference	There were significant improvements in fatigue and quality of life.	The results support for the viability of teleconference-delivered fatigue management education.
Kos et al. [40], 2016, Belgium	To evaluate the effectiveness of an individual SMOoTh vs relaxation on the performance of and satisfaction with relevant daily activities.	31, MS	Fatigue Intervention with the SMOoTh program	There were significant improvements in COPM.	Both interventions showed improvements in the satisfaction and performance of activities.
Lamb et al. [31], 2004, United States	To evaluate the effectiveness of an energy conservation program vs self-study material at home in a missed session	92, MS	Energy conservation program	There were no significant differences between groups.	The self-study material is just as effective if the person miss a session, but it would not work as the only method of treatment.
Hersche et al. [39], 2019, Switzerland	To evaluate the effectiveness of the inpatient energy management education (IEME)	47, MS	Fatigue intervention through IEME program	There were significant improvements in fatigue in both groups. The IEME alone resulted in significant improvements in self-efficacy regarding energy conservation strategies.	The IEME program was effective at improving self-efficacy in performance and fatigue management strategies.

MS: Multiple sclerosis, ALS: Amyotrophic Lateral Sclerosis, IG: Intervention group, CG: Control group, QoL: Quality of life, OT: Occupational therapy, SPT: speed of processing training, vs: versus, SMOoTh: self-management occupational therapy intervention, COPM: Canadian Occupational Performance Measure, IEME: inpatient energy management education.

**Table 3:** Characteristics of the interventions performed in the studies included in this scoping review.

Author, Year, Country	Intervention Category	Intervention	CG/IG	Duration (Weeks)	Sessions	Measurment Instruments	Intervention Manager
Eyssen et al. [55], 2013, Netherlands	Other categories	Client-centred OT	CG: Traditional therapy for the patient.The client-centred framework and tools were not available. IG: OTs encouraged participants to choose, organise and perform activities the patients found useful and meaningful in their environment. The client-centred process model was based on the Canadian practice process framework.	52	*NS*	EDSS, DIP, IPA, 9HPT, MFIS, PES, SF36, COPM, ECGP	OT
Eyssen et al. [56], 2014, Netherlands	Other categories	Client-centred OT	CG: Traditional therapy for the patient. The client-centred framework and tools were not available IG: OTs encouraged participants to choose, organise and perform activities the patients found useful and meaningful in their environment. The client-centred process model was based on the Canadian practice process framework.	104	*NS*	EDSS	OT
Raglio et al. [58], 2016, Italy	Other categories *	Music therapy *	CG: Participants received physical and speech rehabilitation, OT an psychological support IG: Music therapy sessions. OTs stimulates patients to communicate using instruments and express emotions.	4	Three-weekly half-hour sessions	ALSFRS-R, HADS, MQoL-it, MTRS	OT
Block et al. [57], 2009, United States	Other categories	Health-promotion and self-efficacy management	CG: Nontreatment IG: A variety of indoor and outdoor activities for indepent living and health promotion like using public transport or recreational activities included sailing or cycling.	23	Ten full day/sessions, twice a month	GSE, PAL	OT, psichologist
Reilly y Hynes. [42], 2018, Ireland	Cognitive intervention	Cognitive Occupation- Based Programe (COB-MS)	IG: Compensatory strategies and new routines and techniques about employment and daily life. There are seven group session and one individual session. Participants increase their knowledge about cognition, sleep, motivation and future goals. Pre-test/post-test were done.	8	Once-weekly sixty minutes sessions	GAS, OSA-DLS, CVLT-II, BVMT-R, SDMT, TMT, BRIEF-A, EMQ-R	OT
Chiaravalloti et al. [43], 2018, United States	Cognitive intervention	Speed of Processing Training (SPT)	CG: Nontreatment IG: Three tasks about speed of processing, divided attention and selective attention on a computer. First, participants practice a discrimination task with targets. In task 2, participants have to locate a peripheral target while they are doing task 1. In task 3, they have to do the same at task 2 but with distracters.	5	Twice-weekly thirty to forty minutes sessions	WAIS-III, LC, PC, CVLT-II, TIADL	OT, neuropsychologists
Goverover et al. [20], 2017, United States	Cognitive intervention	Self-generation learning program (self-GEN trial)	CG: Memory and learning tasks. Participants have to learn an items list. IG: Memory and learning task with techniques to improve this skills. Participants have to learn the same list but it has pictures, sentences or a word pair. Participants can choose whose the most useful technique to learn words is.	3	Twice-weekly sixty minutes sessions	CMT, SRSI, MIST, CVLT-II, MFQ, AQ, FBP, CMDI, FAMS	OT
Schettini et al. [41], 2015, Italy	Cognitive intervention *	Assistive technology prototype for communication and home control *	IG^1^: Participants were asked to control a standalone P300-speller based BCI to test the ability to control a BCI system and to subsequently compare the performance obtained with the BCI with that observed while controlling the assistive technology prototype with the BCI channel. IG^2^: Users, who operated via a conventional or an alternative input device (eg, mouse, buttons) that best matched their residual motor abilities controlling the assistive technology. Two task: Self-managed environmental control task: and Self-managed communication task. IG^3^: Assistive technology. The prototype visual interface consisted of several menus. Stimulation timing and number of stimulus repetitions for each item were the same as in condition Two tasks: Copy environmental control task: and Copy communication task.	3	Once-weekly ninety minutes session	BCI online copy accuracy, BCI offline accuracy, AT prototype online accuracy during self managed tasks). BCI offline Writen Symbol Rate, AT prototype time for correct selection). VAS, System Usability Scale.	OT, engineer, neurologist
Gentry. [19], 2008, United States	Cognitive intervention	0T therapy using PDAs as assistive technology	IG: Participants learn to use PDAs for three weeks (week 10 to 12) and the OT measure eight weeks before and eight weeks later. They learn about calendar reminders, use of contacts, troubleshoot and train in use of additional features.	21	Two sixty minutes sessions and two ninety minutes sessions	RBMT-E, COPM, CHART-R	OT
Shevil et al. [44], 2009, Israel-United States	Cognitive intervention	Program: Mind over Matter.knolwedge and management.	IG: OTs teach to participantes about cognitive impairments and how to manage their symptoms. about how increase participant´s self-efficacy and the use of cognitive strategies.	5	Once-weekly two hours sessions	Knowledge quizzes, CMSEQ, CSQ	OT
Gómez-Fernández et al. [54], 2001, Cuba	Physical intervention *	Multifactorial intervention *	IG: Participants received a multifactorial treatment with breathing exercises; face, mouth and neck exercises, balance and walk exercises. OTs have to avoid participants fatigue in the treatment. Pre-test/post-test.	4	Monday to Friday seven hours’ sessions. Saturday three hours sessions	FVC, ALSFRS	OT, Neurologists, physiotherapists, logopaedits, defectologists, psychologists and physicians
Yang et al. [53], 2019, United States	Physical intervention	ActiveStep treadmill to improve stability and falls risk	IG: Participants walk on the treadmill with a safety harness and they have to adapt to unexpected slips.		Five sessions	Number of falls, COM, quality of steps	OT, kinesiologist, mathematical
Kamm et al. [46], 2014, Switzerland	Physical intervention	A home-based program to improve manual dexterity in ADL	IG1: Participants are in two randomized groups. The first group practice a dexterity program (finger tapping, turning coins, modeling clay). IG2: The second group practice a theraband program with strength exercises. Pre-test/post-test.	4	Five weekly thirty minutes sessions	CRT, NHPT, JAMAR, CAHAI,	OT, neurologist
Lamers et al. [45], 2019, Belgium	Physical intervention	A task-oriented program to upper limb	CG: Conventional occupational therapy IG: Participants train the task-oriented program at individualized intensity. They have to practice unilateral and bilateral tasks in their daily life and the difficulty is increased throughout the program.	8	Five weekly sixty minutes sessions	NHPT, ARAT, BBT, TEMPA, MAM-36	OT
Finlayson et al. [52], 2009, United States	Physical intervention	“Safe at Home BAASE” a fall risk management program.	IG: Participants train the program to increase the knowledge about falls and to learn skills to manage falls. There are 14 fall prevention strategies. In post-intervention, participants report whether they use the strategies. Pre-test/post-test.	6	Once weekly two hours sessions	FCS, FMS, FPMQ, FPSS, FES	OT
Ortiz et al. [50], 2013, Spain	Physical intervention	A virtual reality rehabilitation to improve balance and postural control	CG: Participants received physiotherapy treatment with strength exercises, propioception exercises, gait facilitation and muscle-tendon stretching. IG: Participants received telerehabilitation treatment using the Xbox 360^®^ console monitored via videoconference following activities that have a certain difficulty and intensity (hitting object with hands and feet, imitating postures, obstacles).	10	Four weekly twenty minutes sessions	CDP, SOT, MCT	Multidisciplinar: physiotherapist/ OT
Waliño-Paniagua et al. [47], 2019, Spain	Physical intervention	OT virtual reality compared to conventional OT	CG: Conventional occupational therapy. IG: Participants received OT and virtual reality include leisure activities (play cards, play hockey, fishing)	10	Twice weekly thirty minutes sessions	PPT, JPT, GPT	OT
Bovend´Eerdt et al. [51], 2010, United Kingdom	Physical intervention	An integrated motor imagery program	CG: Participants watch a film with physical practice, Then, They have conventional OT and physiotherapy. IG: Participants watch a different film than CG and then, OTs train with patients with imagery strategies in particular tasks.	7	Two to three weekly six hours and a half the total time spent	GAS, BI, RMI, ARAT, NEADLS	OT, Physiotherapy
Kalron et al. [48], 2013, Israel	Physical intervention	A sensory re-education program on hand sensibility and manual dexterity	CG: Participants received OT sessions with non specific exposure via grasping objects. IG: Participants received two tasks. In the first task, participants are blindfolded and they have to recognize the object. In the second task, all objects are on a table and OTs describe the object. They have to discriminate it.	10	Five weekly twenty minutes sessions.	NHPT, FDT, TDP, S-W monofilaments	OT
Bansi et al. [49], 2013, Switzerland	Physical intervention	Effects of a endurance training in quality life and fatigue	IG: Participants in two groups performed a 3 weeks endurance exercise training on a cyclo-ergometer or an aquatic bike with different phases.	3	Four daily thirty to forty minutes sessions	FSMC, MFIS, SF-36	OT, physiotherapist, neurophysicologist
Vanage et al. [29], 2003, United States	Fatigue intervention	An energy conservation course	CG: Participants received 8 weeks control treatment and then, they received 8 weeks energy conservation course. IG: Participants received 8 weeks energy conservation course and then, they received 8 weeks control treatment.	8	Once weekly sixty minutes sessions	FSS, FIS, MCA	OT
Rietberg et al. [36], 2014, Netherlands	Fatigue intervention	Multidisciplinary rehabilitation on chronic fatigue	CG: Participants received nurse consultation IG: Participants received physiotherapy, OT or social work sessions when they need. Physiotherapy sessions were determinate in 45 min sessions.	12	Number of sessions was on an as-needed basis, with a mínimum of 2 sessions	CIS-20R, MFIS FSS, FIM, DIP, IPA, MSIS-29, SF-36	OT, physiotherapy, social worker
Mathiowetz et al. [30], 2001, United States	Fatigue intervention	An energy conservation course on fatigue impact	CG: Participants received 6 weeks control intervention with support and discussing about MS topics. IG: Participants received the energy conservation course learning about rest, communication, ergonomic principles, activity and balance lifestyle.	6	Once weekly two hours sessions	FIS, SEG, SF-36	OT
Ghahari et al. [34], 2009, Iran	Fatigue intervention	An online fatigue self-management program	IG: Reachers transform the Energy Conservation Course into a online self-management program through sharing stories, information and activities. With an online version, patients can practice the program at home.	6	Once weekly two hours sessions	PW-BI, FIS, ACS, FSS, GES, DASS	OT
D´hooghe et al. [36], 2018, Belgium	Fatigue intervention	TeleCoach program by smartphone	IG: Participants received in their smartphones motivational messages focusing on energy management and monitoring the physical activities to improve fatigue levels.	12	NS	FSMC, MFIS	OT, neurologist, neuroscientist
Kos et al. [38], 2007, Belgium	Fatigue intervention	Multidisciplinary fatigue management program.	CG: Participants received information about topics that did not concern to fatigue (car adaptation, communication skills or general information abpur MS) IG: Participants received information about pharmacological treatment, diet, rest, strategies to manage fatigue or adaptation to work or home.	4	Once weekly two hours sessions	MFIS, FSS	OT, Multidisciplinary team
Sauter et al. [35], 2008, Austria	Fatigue intervention	A course of energy conservation for people with MS	CG: Participants did not receive treatment IG: Participants received information about different topics like rest, self care, communication, work or leisure tasks.	6	Once weekly two hours sessions	FSS, MFIS, MS-SFS, EDSS, MSFC, PSQI S-RSD	OT
Mathiowetz et al. [32], 2005, United States	Fatigue intervention	A course of energy conservation for people with MS	CG: Participants received 6 weeks control intervention with support and discussing about MS topics. IG: Participants received the energy conservation course learning about rest, communication, ergonomic principles, activity and balance lifestyle.	6	Once weekly two hours sessions	SEG, FIS, SF-36	OT
Finlayson et al. [33], 2011, United States	Fatigue intervention	A teleconference- delivered fatigue management program for people with MS	IG: Participants were divided in two groups and they received the treatment in different weeks by teleconference. The intervention consist in teaching sessions, discussing and homework about topics like communication, fatigue, rest, ergomonics and balanced life.	6	Once weekly seventy minutes sessions	SF-36, FIS, FSS, SECQ	OT
Kos et al. [40], 2016, Belgium	Fatigue intervention	A self-management fatigue program (SMOoTh)	CG: Participants received physiotherapy sessions with relaxing techniques and some information. IG: Participant received information about fatigue, levels of activity, communication, use of wheelchairs, obstacles and facilitators at home and some strategies.	3	Once weekly thirty to ninety minutes sessions	SF-36, MFIS, COPM, CIS-20R,	OT
Lamb et al. [31], 2004, United States	Fatigue intervention	Energy conservation.	CG: Conventional OT. Participants did not receive any modules of treatment IG: Participants divided in three groups and they received one module, two modules, or more than two modules. Evaluators tried to check the course efficacy if participants do not receive some sessions.	6	Once weekly two hours sessions	SF-36, FIS, SEA, ECSS	OT
Hersche et al. [39], 2019, Switzerland	Fatigue intervention	An energy management education program.	CG: Participant received progressive muscles relaxation or group sessions. IG: Participants discussed and work about topics such as occupational balance, activity, fatigue, energy account, goals or effective communication.	3	Once weekly Two hours sessions	MFIS, SF-36, UWSES, SEPECSA	OT

* ALS intervention; OT: Occupational therapy; OTs: Occupational therapist; NS:Not stated; BCI: Brain computer interface; ACS: Activity Card Sort; ALSFRS: Amyotrophic Lateral Sclerosis Functional Rating Scale; AQ: Awareness Questionnaire; ARAT: Action Research Arm Test; BBT: Box and Block Test; BI: Barthel Index; BRIEF-A: Behavior Rating Inventory of Executive Function; BVMT-R: Brief Visuospatial Memory Test-Revised; CAHAI: Chedoke Arm and Hand Activity Inventory; CDP: Computerized dynamic posturography; CHART-R: Craig Handicap Assessment and RatingTechnique-Revised; CIS-20R: Checklist Individual Strength; CMDI: Chicago Multiscale Depression Inventory; CMSEQ: Cognitive Management Self-Efficacy Questionnaire; CMT: Contextual Memory Test; COM: Center Of Mass; COPM: Canadian Occupational Performance Measure; CRT: Coin Rotation Task; CVLT-II: California Verbal Learning Test–2nd Edition; CSQ: Cognitive Strategies Questionnaire; DASS: Depression Anxiety and Stress Scale; DIP: Disability and Impact profile; ECGP: Evaluation of the Client-Centered Process; ECSS: Energy Conservation Strategies Survey; EDSS: Expanded Disability Status Score; EMQ-R: Everyday Memory Questionnaire-Revised; FAMS: Functional Assessment of Multiple Sclerosis; FBP: Functional behavior profile; FCS: Falls Control Scale; FDT: Functional Dexterity Test; FES: Falls Efficacy Scale; FIM: Functional Independence Measure; FIS: Fatigue Impact Scale; FMS: Falls Management Scale; FPMQ: Falls Prevention and Management Questionnaire; FPSS: Fall Prevention Strategies Survey; FSMC: Fatigue Scale of Motor and Cognitive Functions; FSS: Fatigue Severity Scale; FVC: Forced Vital Capacity; GAS: Goal Attainment Scaling; GES: Generalized Self-Efficacy Scale; GPT: Grooved Pegboard Test; HADS: Hospital Anxiety and Depression Scale; IPA: Impact on Participation and Autonomy; JTT: Jebsen- Taylor Hand Function Test; LC: Letter Comparison; MAM-36: Manual Ability Measure; MCA: Measure Change Assessment; MCT: Motor Control Test; MIST: Memory for Intentions Test; MFIS: Modified Fatigue Impact Scale; MFQ: Memory Functioning Questionnaire; MQoL-it: Italian version of McGill Quality of Life Questionnaire; MSFC: Multiple Sclerosis Functional Composite; MS-SFS: Multiple Sclerosis- Specific Fatigue Scale; NEADLS: Nottingham Extended Activity of Daily Living Scale; NHPT: Nine Hole Peg Test; OSA-DLS: Occupational Self-Assessment-Daily Living Scales; PAL: Personal Activity Log; PC: Pattern Comparison; PES: Pain Effects Scale; PPT: Purdue Pegboard Test; PSQI: Pittsburgh Sleep Quality Index; MSIS-29: Multiple Sclerosis Impact Scale; PW-BI: Personal Well-Being Index; RBMT-E: Rivermead Behavioral Memory Test- Extended; RMI: Rivermead Mobility Index; SDMT: Symbol Digit Modality Test; SEA: Self-Efficacy for Performing Energy Conservation Strategies Assessment; SECQ: Self-efficacy for Energy Conservation Questionnaire; SEG: Self-Efficacy Gauge; SEPECSA: Self-Efficacy for Performing Energy Conservation Strategies Assessment; SOT: Sensory Organization Test; S-RSD: Self- Rating Scale for Depression; SRSI: Self-Regulation Skills Interview; SF-36: Study Short-Form Health Survey; TEMPA: Test d’Évaluation des Membres Supérieurs des Personnes Âgées; TDP: Two Discrimination Points; TMT: Trail Making Test; TIADL: Timed Instrumental Activities of Daily Living Test; UWSES: University of Washington Self-Efficacy Scale; VAS: Visual Analog Scale; WAIS-III: Wechsler Adult Intelligence Scale-III. Session duration is reported where available.

### 3.4. Other Interventions

Four articles did not fit into any of the previous categories. Of these, three studies focused on MS patients.

Two of the studies focusing on MS assessed client-centered practice intervention in people with MS patients to evaluate disability, autonomy and participation in daily life with no significant effects in these outcomes [55,56]. Another of these studies, led by Block et al., assessed the effectiveness of health promotion in people with MS which worked on different aspects such as the empowerment of the person [57]. This study showed significant improvements in self-efficacy and ability to achieve objectives [57].

The one study focusing in ALS evaluated the impact of music therapy programs on psychological aspects such as depression and anxiety [58]. In this study, occupational therapists stimulates patients to communicate using instruments and express emotions with positive results in quality of life.

## 4. Discussion

This scoping review describes different occupational therapy interventions carried out in MS and ALS patients. These interventions were mainly focused on physical rehabilitation, cognitive rehabilitation and reducing fatigue. Although some of the interventions included in this review were not exclusively led by occupational therapists, they can use these interventions to facilitate the occupational therapy evidence-based interventions. This review shows that the majority of occupational therapy interventions are performed on MS patients while there is little information about ALS patients. In fact, few intervention studies led by occupational therapists have been found.

### 4.1. Fatigue Interventions and Energy Conservation

This study shows that certain occupational therapy interventions for MS and ALS patients could be effective in improving different outcomes. The majority of the studies identified in this scoping review were fatigue interventions carried out in MS patients. In our search we found that studies principally focused on fatigue are based on the Packer et al. program [28]. This fatigue program is a six-week energy conservation course, which was designed for adults suffering from fatigue as a symptom of chronic disease [28]. In this program occupational therapists educated participants in the benefits of breaking up high-energy tasks by incorporating rest periods into their daily activities [28]. In addition, we observed several studies that included the Packer et al. energy conservation course with some adaptations. Lamb et al. found that patients using self-study material in nonpresential sessions, and who had missed some sessions, obtained similar benefits regarding energy and fatigue management to those whose sessions were guided by a professional and who fully completed the intervention [31]. Similarly, Sauter et al., adapted the fatigue management program to the German population and showed improvements in users´physical and mental fatigue. Subsequent studies [33,34] modified the Packer et al. fatigue management course [28] so that it could be delivered by teleconference and online for people with MS who had problems accessing treatment, leading to an improvement in fatigue and quality of life [33,34]. The therapy showed significant improvements in fatigue management even when participants were guided via technological devices [29,30,31,32,33,34,35]. In a similar way, D´hooghe et al., developed a course related to a fatigue management program using a smartphone to provide monitoring, motivational messaging and the establishment of objectives [36]. The results showed that this type of intervention can be complementary to conventional treatment to reduce fatigue [36]. Overall, according to the previous evidence, the use of new technologies seems to be a good treatment option.

Conversely, other studies explored fatigue management using different multidisciplinary interventions related to personal care. Rietberg et al. evaluated an intervention carried out by multidisciplinary professions including physical therapy, social work and occupational therapy which applied fatigue management strategies and personal care as compared with only nurse consultation and found that multidisciplinary rehabilitation did not lead to a more effective reduction of self-reported fatigue [37]. In the same line, Kos et al. evaluated a multidisciplinary fatigue management program intervention comparing it with an intervention program based on sleep advice and relaxation exercises [38]. It should be pointed out that neither of the two multidisciplinary interventions explored by these authors showed significant results. This could be because chronic fatigue does not improve significantly over time in MS patients only with personal care advice [37]. All interventions were conducted solely by occupational therapists, except for the multidisciplinary interventions and the D´hooghe et al. program [36], in which technology experts collaborated with occupational therapists.

Other authors have proposed other intervention programs [39,40] based on changes in daily occupational performance such as rest management and the proposal of strategies in relation to the management of instrumental activities such as childcare or shopping, that suggest significant improvements in performance, perceived fatigue and individual satisfaction [39,40].

It must be emphasized that fatigue is one of the most frequently reported symptoms in MS patients and can affect their occupational performance [59]. In this sense, a recent review [60] showed that patient-reported outcomes (PROs) are increasingly used in MS treatment. PROs not only describes symptoms, function and health status in MS patients but also evaluates the impact of this disease and assess the concerns on MS patient´s life [60].

Fatigue intervention in the included studies was found to be effective in reducing fatigue, managing fatigue symptoms and improving different aspects such as health-related quality of life [12,29,30,31,32,33,34,35,39,40]. Thus, it is essential that occupational therapy interventions should include fatigue intervention in daily practice with MS and ALS patients.

### 4.2. Cognitive Interventions

With regard to cognitive interventions, we identified six studies in MS and ALS treatment of which three were conducted exclusively by occupational therapists. In MS interventions, Tony Gentry et al., evaluated a program with PDAs that resulted in an improvement of the person´s functional performance and satisfaction using PDA as a compensation for cognitive deficits [19]. The remaining cognitive interventions [20,42,43,44] were related to improving memory, attention, processing speed and strategies to compensate this deficit. Among them, Goverover et al., evaluated the effectiveness of a cognitive strategies program, through visual supports when memorizing words, which showed improvements in memory, learning, depressive symptomatology and quality of life [20]. Only one cognitive intervention was identified for ALS. Schettini et al., evaluated the reliability of an assistive technology device for home automation control and communication, and there were no significant improvements. This could be due to the fact that the sample included only eight people, which may be too small to provide strong evidence [41].

Overall, although cognitive interventions in MS and ALS have scarcely been analyzed, these studies show that cognitive interventions in this type of population have significant beneficial effects in functional performance, depression and quality of life [12,19,20,42,43,44]. However, these results should be interpreted with caution because the samples in most of the studies described were small and there is no evidence regarding their long-term effects on functional performance [41,42,43]. In addition, it also should have taken into account that there is a lack of information about the effectiveness of these interventions in the progressive forms of MS [61], and there is no evidence about therapeutic intervention to enhance cognitive performances in MS patients [62]. Thus, more studies are needed.

### 4.3. Physical Interventions

Evidence based on different physical therapeutic modalities suggested that interventions improve different functional outcomes (manual dexterity), reduce fatigue and improve quality of life [12,23]. All the studies focused on upper limb recovery were carried out in MS patients. Lamers et al., evaluated the ideal intensity in an upper limb recovery program, showing a positive result, although no overall intensity was established [45]. Kamm et al., conducted a program to improve manual dexterity with exercises using fingers, coins, paper and pencil, and clay, showing improvements in fine motor skills in the experimental group [46]. In the same way Waliño-Paniagua et al., evaluated manual dexterity with virtual reality games in comparison with conventional occupational therapy, showing significant differences. These interventions could also be used as complementary activities in occupational therapy [47]. Finally, Kalron et al., conducted a sensory re-education with tubes of different textures and thickness, showing an improvement in manual dexterity and, although sensitivity did not improve, this program may help to prevent deterioration in manual dexterity in early stages of rehabilitation [48]. It should be pointed out that evidence-based rehabilitation for upper limb recovery are essential for improving performance in daily tasks [23]. Thus, occupational therapists could carry out this evidence-based intervention in MS and ALS patients.

With respect to physical rehabilitation interventions, Bansi et al., evaluated physical rehabilitation with cycle-ergometers or aquatic- bikes in two groups, showing an improvement in quality of life and fatigue [49]. Another study examined virtual reality rehabilitation with strength and proprioception exercises on unstable surfaces and muscle-tendon stretching, showing significant improvements at the motor level, which suggests that it could be an alternative treatment [50]. In the same line, Bovend´Eerdt et al., assessed a film and image presentation program with exercise information and guided rehabilitation strategies [51]. This intervention did not provide valid results because participants did not perform the program in the established time, although there were significant differences after the intervention [51].

We only identified one study in ALS patients, where Gómez-Fernández et al., examined a multifactorial program in ALS, through postural control exercises, exercises with lips, breathing, walking or psycho-emotional support that showed significant improvements in forced vital capacity [54]. However, the sample is very small, which could cast doubt on the results [54].

Regarding the interventions aimed at falls prevention, previous studies carried out programs either by receiving information about falls, strategies and changes in the environment [52], or by treadmill with caused imbalance [53]. Both studies showed a decrease in falls in MS patients.

### 4.4. Other Interventions

Evidence for other interventions was limited. We included four articles in this category because they did not fit into any of the previous categories. In this category, Eyssen et al., explored the effectiveness of client centered practice, comparing it with a control group that received conventional occupational therapy practice [55,56]. The results showed no significant improvements in participants, possibly because more time was spent on evaluation than on intervention, resulting in a less effective recovery. Therefore, this type of practice is not recommended [55,56].

There is currently only limited evidence for the effectiveness of the role of environment in the experience of disability. However, the project Shake-it-up explored the effectiveness of health promotion which works on aspects such as self-efficacy and empowerment among others and found a significant improvement in these aspects [57]. These results could be useful for occupational therapists in their routine work in order to improve the independence, community access and participation of MS and ALS patients.

Finally, we also found one study which assessed the impact of music therapy in ALS, participants interacted with different instruments to express their emotions and communicate, showing an improvement in their quality of life [58]. These findings suggest that there is a need for better designed intervention studies which explore the impact of music therapy on other symptoms in ALS and MS patients.

### 4.5. Study Limitations

This study has a number of limitations. First, regarding inclusion criteria, we only included studies published in English or Spanish and those with full text available. Second, the articles included in this review were experimental studies and might contain biases associated with the experimental study design. Furthermore, the heterogeneity of the included studies meant that they were not comparable in terms of sessions, hours and study objectives. The generally limited study sample size of some included studies means that the results should be interpreted with caution. Third, like other authors in their respective scoping reviews, we did not critically assess the quality of the included studies, because this is not the role of a scoping review [63]. However, we mentioned the limitations of some of the studies in the discussion section. Finally, it should be pointed out that some studies did not clearly specify which professionals participated in the intervention or what their role in the study was. Thus, more studies are needed that specify the role of the researchers in the interventions, including those which are led by occupational therapists.

However, this review also has several strengths. To the best of our knowledge, it is the first study with the aim of describing the main occupational therapy interventions carried out in MS and ALS. In addition, this scoping review highlights the gaps in our knowledge: (i) there is no evidence regarding occupational therapy interventions carried out in Spain; (ii) most of the studies had small sample sizes and a lack of randomization; (iii) there is little evidence about long-terms interventions; and (iv) there is a need to determine the role of the different professionals in the multidisciplinary teams. These identified gaps of knowledge might be dealt with in future research.

This study provides the professionals with a description of therapies in MS and ALS that can support the use of early therapeutic interventions aimed at optimizing outcomes in this population. The included studies in this review showed that occupational therapists can not only collaborate in the multidisciplinary intervention but can also lead different interventions in MS and ALS. This review suggests that occupational therapy is a relevant discipline for MS and ALS patients’ rehabilitation. The main intervention led by occupational therapists is fatigue management, which showed beneficial effects in MS patients, but occupational therapists could also carry out psychosocial, physical and emotional interventions in this population. In addition, we would like to underline that the updated summary of previous evidence carried out in this scoping review provides knowledge to facilitate occupational therapy evidence-based interventions.

Finally, our findings add new insights about the potentially beneficial role of physical rehabilitation, fatigue and cognitive interventions, and could inform future evidence-based guidelines for ML and ALS patients.

## 5. Conclusions

In conclusion, most studies were conducted in the MS population, with little representation from the ALS population. The main interventions in occupational therapy were those aimed at fatigue, cognitive interventions and physical rehabilitation. These interventions have shown an improvement in perceived fatigue, manual dexterity, falls prevention and in cognitive aspects such as memory, communication, depression and quality of life in the MS and ALS population. It should be pointed out that some of the interventions included in this review are not exclusive to occupational therapy practice. However, occupational therapy professionals can use these interventions in patients with MS and ALS, and they can help patients to incorporate activities and occupations into their intervention patterns.

## Figures and Tables

**Table 1:** Database and search strategy.

Database	Strategy
Pubmed	“occupational therapy” [All Fields] AND (“methods” [MeSH Terms] OR “methods” [All Fields] OR “intervention” [All Fields]) AND “sclerosis” [All Fields]
Scopus	TITLE-ABS-KEY (“occupational therapy” AND intervention AND “sclerosis”)
Embase	(‘occupational therapy’/exp OR ‘occupational therapy’) AND (‘intervention’/exp OR intervention) AND (‘sclerosis’/exp OR sclerosis)
Web of Science	(“occupational therapy” AND intervention AND sclerosis)
Teseo	(“occupational therapy” AND intervention AND sclerosis)
Journal of Occupational Rehabilitation	‘“occupational therapy” AND intervention AND sclerosis’
Physical & Occupational Therapy In Pediatrics	[All: “occupational therapy”] AND [All: intervention] AND [All: sclerosis] AND [in Journal: Physical & Occupational Therapy In Pediatrics]
American Journal of Occupational Therapy	“occupational therapy” AND intervention AND sclerosis
Occupation, Participation and Health	[All “occupational therapy”] AND [All intervention] AND [All sclerosis]

## Data Availability

All data is presented in this article. Researchers can contact authors regarding any request about the data.
